# The relationship between serum 25-hydroxy vitamin D and blood pressure and quality of life in overweight and obese patients with type 2 diabetes mellitus compared with healthy subjects

**DOI:** 10.22088/cjim.11.3.267

**Published:** 2020-05

**Authors:** Naser Aghamohammadzadeh, Neda Dolatkhah, Maryam Hashemian, Seyed Kazem Shakouri, Saeed Hasanpour

**Affiliations:** 1 Endocrine Research Center, Tabriz University of Medical Sciences, Tabriz, Iran; 2 Physical Medicine and Rehabilitation Research Center, Aging Research Institute, Tabriz University of Medical Sciences, Tabriz, Iran; 3 Department of Biology, School of Arts and Sciences, Utica College, Utica, United States; 4 Faculty of Medicine, Tabriz University of Medical Sciences, Tabriz, Iran.

**Keywords:** 25-hydroxyvitamin D, Diabetes mellitus, Blood pressure, Quality of life

## Abstract

**Background:**

Vitamin D is one of the known lipoprotein hormones with metabolic properties. We aimed to determine the serum 25-hydroxy vitamin D concentration in overweight/obese subjects with diabetes mellitus type Ⅱ (DM Ⅱ) in association with systolic and diastolic blood pressure and quality of life compared with healthy participants.

**Methods:**

The current case-control study was carried out among 80 overweight/obese subjects with DM Ⅱ, and 77 healthy subjects matched by sex, age and body mass index (BMI). Serum 25-hydroxyvitamin D concentration was measured by ELISA method. In order to examine the quality of life, the Persian version of SF36 questionnaire was used.

**Results:**

There was significant difference between diabetic and healthy subjects considering serum 25-hydroxyvitamin D concentration (p=0.012). Serum 25-hydroxyvitamin D concentration was inversely correlated with diastolic blood pressure (p=0.02) and positively associated with physical function (p<0.001), social function (p<0.001) and general health (p<0.001) components of quality of life in diabetic subjects and physical health sub-scale (p=0.004) in all participants.

**Conclusion:**

Serum 25-hydroxyvitamin D concentration was significantly lower in diabetic subjects in comparison with healthy controls. There was a significant reverse relationship between serum concentrations of 25-hydroxyvitamin D with diastolic blood pressure and on the other hand, a significant positive relationship with physical function, social function and general health components and physical health subscale of quality of life in participants with DM Ⅱ.

In recent years, several non-communicable diseases (NCDs) have been described to be linked with vitamin D deficiency including diabetes mellitus (DM) (1, 2). DM is one of the most common NCDs in the developing countries, and has the fastest growth globally in the current decades (3, 4). Vitamin D is known as one of the lipoprotein hormones, which, apart from the major effects on calcium and phosphorous homeostasis, has many known effects in the human body. Some evidence is available that adequate concentrations of 25-hydroxyvitamin D may have protective effects on the metabolic syndrome development (5). Vimaleswaran KS *et al*. have suggested that higher serum concentration of vitamin D is associated with a decreased systolic blood pressure and lower risk for hypertension (HTN) (6). Iran is one of the areas that seems to have a high prevalence of vitamin D deficiency. It is estimated that about 70% of the Iranian population suffer from vitamin D deficiency/ insufficiency (7). More than 80% of diabetic patients are obese and prone to HTN (8, 9). 

Overweight and obesity are important risk factors for vitamin D deficiency, and a recent observational study in the general population has shown a negative correlation between the vitamin D deficiency and blood pressure measured in outpatient department (10). On the other hand, the probable significance of vitamin D deficiency and its role in quality of life and other aspects of health have attracted a great interest in studies of healthy people and those with chronic diseases (11-19). However, few investigations have examined the effect of vitamin D deficiency on the health associated quality of life in diabetic patients (20, 21).

Considering the importance of vitamin D in regulating blood pressure, as well as insulin resistance, and the contradiction between the results of the published studies, the aim of this investigation was to measure the serum concentration of 25-hydroxyvitamin D in overweight/obese subjects with DM Ⅱ in association with systolic and diastolic blood pressure and quality of life compared with healthy subjects. 

## Methods

This is a case-control study. The target population of the study was all patients with DM Ⅱ in Tabriz city. Cluster-random sampling was carried out in the health centers of Tabriz. Tabriz has 82 health centers in 10 different municipal districts, with the characteristics of all diabetic patients including phone number and address at the centers. First, 5 municipal districts were randomly selected (1, 3, 5, 6 and 9). Thereafter, two health centers in each area were randomly selected and then investigators went to the health centers (10 centers) and extracted the list of all patients along with their phone numbers and addresses, and then the patients were selected via relative randomness approach (the number of diabetic patients covered by each health center) (Flowchart 1), then they were contacted and requested to participate in an orientation meeting in one of the health centers. In the meeting, a brief description of the aims and the method of the study were clarified, and at the same time the criteria for the study were examined. 

Inclusion criteria were age 20 and above, diagnosis of DM Ⅱ and body mass index (BMI) of 25 kg/m2 and above. Exclusion criteria were type 1 diabetes, previous hypertension or antihypertensive drug use, taking any dietary supplement including vitamin D supplementation, following special diets (vegetarianism, etc.), history of known liver or kidney disease, the presence of malignancy or the history of recent acute illnesses, pregnant and lactating women, history of taking medications affecting vitamin D levels, taking corticosteroids, anticonvulsants, estrogen, androgen and oral contraceptive (OCP), history of pancreatitis, hypo- or hyperthyroidism, Cushing’s disease, Addison’s disease and renal failure. The control group was randomly selected from referrals to health centers without known history of illness matched by age, sex and BMI with a 1:1 ratio. Exclusion criteria for control group were blood glucose level ≥ 120 mg/dl and HbA1c of 6.5% and higher (22).

Sample size: Considering the level of blood pressure in the case and control groups as the primary outcome, and considering the observations in the Nayak study (23), 24.2 and 20.0 were respectively the standard deviation (SD) of the case and control group and 8.8 for the effect size. With a significant level of 0.05, power of 0.7 and through a two-way test, the sample size was computed as 77 in each group. Considering 10% for the probability of losing, sample size in each group was 85 and in total 170 individuals.

If the conditions were met, written informed consents were obtained and the demographic and physical activity questionnaires and anthropometric checklist were completed. Dietary intake: In order to investigate the food intake, a 3-day food record was obtained and macro- and micro-nutrients intakes were determined through U.S. Department of Agriculture (USDA) food composition (24) with changes to Iranian foods. Since the Iranian food composition tables are not thorough and contain inadequate data on raw foods and beverages (25), the Department of Agriculture (USDA) food composition tables were applied to determine the content of energy and nutrients in foods that the Iranian food composition tables were not completed (26).

Anthropometric measurement: Participants’ weight was evaluated by a standard scale (Seca 813 digital scale) following an overnight fasting. Participants’ height was evaluated by a standard scale (Seca 206 roll-up measuring tape). BMI was computed through dividing the weight (kg) by the square of height (m2). 

BMI between 18.5 and 24.9 has been defined as normal. BMI values greater than 25 and 30 are regarded as overweight and obese, respectively (27). Physical activity: Participants’ physical activity was evaluated by the Persian version of International Physical Activity Questionnaire (IPAQ) (28-30). Three categories were defined: low, moderate, and high physical activity (31). 

**Figure:**
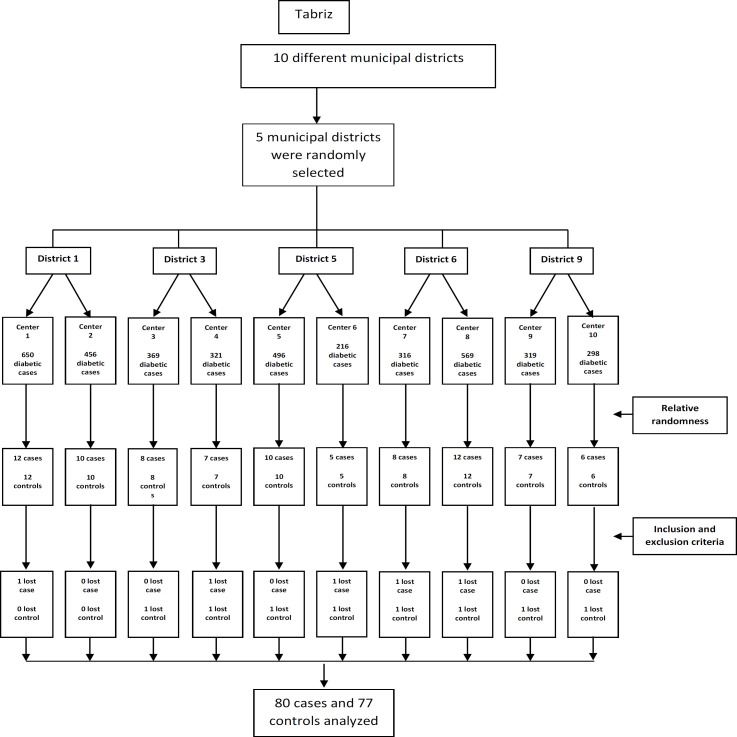
Flowchart of participants

Vitamin D measurement: Fasting blood samples were collected. Serum 25-hydoxyvitamin D concentrations were measured in the laboratory of Emam Reza Hospital in Tabriz as a reference laboratory, following all the essential strategies for blood sampling and prohibition of blood hemolysis during collection. Blood samples were rapidly centrifuged at 3500 rpm for 10 to 15 minutes to isolate the serum. Samples were stored at -80 ° C till the laboratory tests were carried out. To minimize the error, the measurement of each biochemical parameter was done by one individual, one kit and one specific device. Serum 25-hydroxyvitamin D concentrations were admeasured by ELISA. 

All stages were implemented based on the directions of the kits (Euroimmun, Germany). Experimental reliability was investigated by sending 5 duplicates with two different names. Individuals were divided into three groups of deficient (0-19.9 ng/ml), insufficient (20-29.9 ng/ml) and sufficient (≥ 30 ng/ml) groups based on 25-hydroxyvitamin D concentration (32).

Blood pressure measurement: The blood pressure of participants was measured in the sitting position from the right arm of the subjects by a Riester manometer, Diplomat model with accuracy of ±3 mm Hg after 15 minutes of rest per visit. Measurements were executed twice, with interval of 15 minutes. Quality control of the device was performed every month. It was recommended that people should not perform intense exercises, eating, smoking, drinking except water and using drugs that affect blood pressure, for at least 1 hour before measuring blood pressure. The cuff was 12-12.5 cm in width and the height was at least two-thirds of the arm’s circumference. For analysis, the mean of two measurements was used.

Quality of life: The tool used to measure quality of life was the Persian version of the 36-item questionnaire SF36. This questionnaire is valid for assessing the quality of life under various conditions, including diabetes. The validity and reliability have been evaluated and accepted in Iran, and the Cronbach’s alpha has been between 0.77-0.9 for eight domains (33). Statistical analysis: Data are provided as mean (± standard deviation) and frequency (percentages) for quantitative and qualitative variables, respectively. Data analysis has been done by SPSS software version 16 (SPSS Inc., Chicago, IL). 

The normal distribution of data was assessed using the Kolmogorov-Smirnov test and also descriptive evidence such as skewness and kurtosis plus appropriate and reasonable standard deviations (compared to the mean). Chi-square test was applied to compare qualitative variables between the two groups. Independent samples t-test was applied to compare quantitative variables between the two groups. Logistic regression analyses were performed to define whether 25-hydroxyvitamin D concentration was a significant factor correlated with occurrence of HTN. Lastly, partial correlation analyses were applied for analyzing correlations. Age, sex and BMI were considered as control variables. Values of p<0.05 were considered as statistically significant. Ethical considerations: The aims and the method of the investigation were described for each individual at the start of the study, and written informed consent was achieved from each study participant. The participants were able to withdraw the study at any stage during the study, without giving any reason. 

No extra charges, complication or intervention were enforced on participants, and they were not excluded from their routine therapies. This study has been approved by the Ethics Committee of the Research Deputy of Tabriz University of Medical Sciences (IR.TBZMED.REC.1396.823).

## Results

In this study, 80 overweight and obese patients with DM Ⅱ, and 77 healthy subjects matched by age, sex and BMI as control group, were examined. Table 1 shows the demographic characteristics of participants in the two groups of study. Among the participants, 48 (60.0%) of diabetic cases were males and 32 (40.0%) cases were females, compared to 40 (51.9%) and 37 (48.1%) in the control group (p=0.311). In addition, 35 (43.75%) of diabetic cases were overweight (BMI=25.1-29.9 kg/m^2^) and 45 (56.25%) cases were obese (BMI≥30 kg/m^2^), compared to 33 (42.58%) and 44 (57.14%) in the control group (p=0.910). The majority of participants had no or limited education (i.e. 57 (71.2%) subjects in case group and 53 (68.8%) subjects in control group). Physical activity did not differ among cases and controls (p=0302). 

Table 2 shows the statistical indices of dietary intake, serum vitamin D concentration, systolic and diastolic blood pressure, and various components of quality of life in participants. There was no significant difference in the dietary intake of vitamin D between the two groups (p=0.357). Regarding the recommended dose of RDA for vitamin D (600 IU), 5.7% (6 cases) in the case group and 10 (12.9%) in the control group had a dietary intakes of higher than RDA. Serum 25-hydroxyvitamin D concentrations were significantly lower in patients with DM Ⅱ than in healthy controls (p=0.012). Regarding the serum vitamin D status, in the case group 41 subjects (51.3%) had deficiency, 22 (27.5%) subjects had insufficiency and 17 (21.2%) subjects were in sufficient range. In the control group 28 (36.4%) subjects had deficiency, 18 (23.4%) subjects with insufficiency and 31 (40.2%) were in sufficient range. There was a significant difference between the two groups (p=0.015).

**Table 1:** Comparisons of demographic characteristics of participants in the case and control groups

**Variable**	**Group**		**Frequency**	**percent**	**p-value**
Age (yr)	56.91±10.75*				0.115
Sex	Case (n=80)	male	48	60.0	0.311
		female	32	40.0	
	Control (n=77)	male	40	51.9	
		female	37	48.1	
BMI (kg/m^2^)	Case (n=80)	25.1-29.9	35	43.75	0.910
		≥30	45	56.25	
	Control (n=77)	25.1-29.9	33	42.85	
		≥30	44	57.14	
Education	Case (n=80)	illiterate	57	71.2	0.782
		Under Diploma and Diploma	17	21.2	
		college	6	7.5	
	Control (n=77)	illiterate	53	68.8	
		Under Diploma and Diploma	20	25.9	
		college	4	5.1.3	
Occupation	Case (n=80)	Unemployed	54	67.5	0.172
		employed	16	20.0	
		retired	10	12.5	
	Control (n=77)	Unemployed	41	53.2	
		employed	30	39.0	
		retired	6	7.8	
Physical activity	Case (n=80)	mild	60	75.00	0.302
		moderate	18	22.50	
		severe	2	2.5	
	Control (n=77)	mild	47	61.03	
		moderate	26	33.76	
		severe	4	5.19	

* mean±SD BMI: body mass index

**Table 2:** Serum concentrations of 25- hydroxy vitamin D, SBP, DBP and QOL components in participants

**p-value**	**Control (n=77)**	**Case (n=80)**	**Variables**	
0.012	28.08±14.56	22.46±12.87	Serum 25-hydoxy vitamin D (ng/ml)	
0.357	326.61±233.19	298.13±157.35	Dietary vitamin D (IU/day)	
0.087	129.68±12.98	133.81±16.89	SBP (mmHg)	
<0.01	78.78±14.79	95.58±15.26	DBP (mmHg)	
<0.01	85.58±22.13	57.20±20.16	PF	QOL
<0.01	30.62±45.17	7.79±16.35	RP	
<0.01	32.08±40.89	6.49±20.26	RE	
<0.01	80.71±5.30	67.75±20.96	EF	
<0.01	83.48±5.16	72.25±17.35	EW	
<0.01	87.66±4.29	62.18±15.90	SF	
<0.01	38.23±24.46	7.92±4.08	P	
<0.01	89.93±9.47	51.07±20.11	GH	
0.030	191.23±27.12	177.13±49.79	PH	
<0.01	258.35±23.90	234.28±39.48	MH	

DBP: diastolic blood pressure, EF: energy/fatigue, EW: emotional well being, GH: general health, MH: mental health, SBP: systolic blood pressure, P: pain, PF: physical function, PH: physical health, QOL: quality of life, RE: Role limitation due to emotional problems, RP: Role limitation due to physical health, SBP: systolic blood pressure, SF: social function

Table 3 shows the relationship between serum concentrations of vitamin D and the systolic and diastolic blood pressure and quality of life in the participants in the study in different groups. The findings of logistic regression analysis of predictor factors of high blood pressure, showed that subjects with vitamin D deficiency are approximately 2.5 times more likely to be affected by diabetes than subjects with normal vitamin D (OR=2.497, 95% CI: 1.24-5.04). These ORs were about 2.47 (OR=2.460, 95% CI: 1.21-4.50) and 2.24 (OR=2.423, 95% CI: 1.20-4.91), with consideration of weight and BMI as the confounding factors, respectively.

**Table 3:** Correlation between serum 25-hydroxy vitamin D with SBP, DBP and QOL in participants

**Partial correlation result**	**group**	**Variables**
r=-0.156, p-value=0.175	Case	SBP
r=0.024, p-value=0.824	Control	
r=-0.256, p-value=0.024	Case	DBP
r=-0.078, p-value=0.507	Control	
r=0.704, p-value=<0.001	Case	PF
r=0.290, p-value=0.012	Control	
r=-0.129, p-value=0.265	Case	RP
r=0.128, p-value=0.275	Control	
r=-0.164, p-value=0.153	Case	RE
r=0.112, p-value=0.340	Control	
r=0.037, p-value=0.747	Case	EF
r=-0.064, p-value=0.589	Control	
r=-0.077, p-value=0.508	Case	EW
r=-0.156, p-value=0.175	Control	
r=-0.077, p-value=0.508	Case	SF
r=0.050, p-value=0.673	Control	
r=-0.054, p-value=0.639	Case	P
r=-0.119, p-value=0.314	Control	
r=0.406, p-value=<0.001	Case	GH
r=-0.079, p-value=0.504	Control	
r=0.315, p-value=0.005	Case	PH
r=-0.268, p-value=0.021	Control	
r=0.009, p-value=0.941	Case	MH
r=0.108, p-value=0.362	Control	

DBP: diastolic blood pressure, EF: energy/fatigue, EW: emotional well being, GH: general health, MH: mental health, SBP: systolic blood pressure, P: pain, PF: physical function, PH: physical health, QOL: quality of life, RE: Role limitation due to emotional problems, RP: Role limitation due to physical health, SBP: systolic blood pressure, SF: social function

The results of logistic regression analysis of predictor factors of high systolic blood pressure, suggested that people who have vitamin D deficiency will be affected by high systolic blood pressure approximately 1.11 times more likely than those with normal vitamin D, which is not statistically significant (OR=1.11, 95% CI: 0.54-2.28). The results also showed that along with one unit increase in the BMI, the chances of having high systolic blood pressure in subjects will increase by 11% (OR=1.11, 95% CI: 1.03-1.19).

The results of logistic regression to predict high diastolic blood pressure, showed that subjects with vitamin D deficiency develop high diastolic blood pressure approximately 2.3 times more likely than those with normal vitamin D (OR=2.28, 95% CI: 1.11-4.75). The results were approximately 2.2 times higher with consideration of weight (OR=2.23, 95% CI: 1.07-4.65) and approximately 2.2 times higher with consideration of BMI (OR=2.25, 95% CI: 1.09-4.68) as the confounding factors.

## Discussion

According to the findings, 51.3% of diabetic patients had vitamin D deficiency, 27.5% had insufficiency and 21.2% were sufficient, while in healthy controls, these levels were 36.4%, 23.4% and 40.2%, respectively, and there was a considerable difference between the two groups in this regard. Vitamin D deficient subjects were 2.5 times more probable to develop DM Ⅱ in comparison with those with sufficient vitamin D status. Serum vitamin D concentrations and all aspects and sub-scales of quality of life were significantly lower and diastolic blood pressures were significantly higher in diabetic patients compared with healthy controls.

Several investigations have revealed the elevated prevalence of vitamin D deficiency in tropical and subtropical countries, including Iran, Turkey, China, India and Saudi Arabia (34-40). The prevalence of vitamin D deficiency in the populace living in Iran in the period 1990-2010 has increased. Women suffer from vitamin D deficiency more than men annually (41). Recently, the importance of vitamin D in obesity has attracted the attention of the relevant researchers, and it has been confirmed that vitamin D deficiency is correlated with obesity and DM (42), and subjects with obesity and hypovitaminosis D are prone to cardiovascular disease (CVD), and several malignancies (43). Hypovitaminosis D seems to decrease the level of intracellular calcium, thereby reduce the level of insulin secretion and beta cellular dysfunction and, as a result, impaired glucose tolerance (44). At the same time, diabetes is linked with an increased hazard of macrovascular events such as myocardial infarction (MI) and stroke (45). Blood pressure levels in people with DM Ⅱ are moderately higher than other people and increased blood pressure is a recognized risk factor for diabetic patients (46, 47). In line with the findings of our study, in a case-control study in Kermanshah Diabetes Clinic in 1993, the serum 25 hydroxyvitamin D concentrations of 90 patients with DM Ⅱ and 90 healthy subjects of the same age and sex were measured and analyzed. According to the results, serum vitamin D concentrations in diabetic subjects were substantially lower than healthy individuals (6.1±12,4 vs. 18±20.7 ng/ml, respectively (p<0.05) (48). However, given the results of a case-control investigation in the Trinidad and Tobago Republic, there was no noticeable difference regarding the serum concentrations of 25-hydroxyvitamin D among diabetic patients and controls (p=0.139) (22).

According to the findings of our study, there was a noteworthy inverse association between serum concentrations of 25-hydroxyvitamin D with diastolic blood pressure in the patients with DM Ⅱ in contrast to healthy controls. Subjects with vitamin D deficiency developed high diastolic blood pressure approximately 2.3 times more likely than vitamin D sufficient ones.

Hypovitaminosis D has been suggested as a probable risk factor for development of HTN; however, there is a controversy about the blood pressure regulating role of vitamin D. Contrary to the findings of this study, in the investigation of Kashani *et.al* (49) there was no association between blood pressure and serum 25-hydroxyvitamin D concentrations in people over 40 years of age. Nayak et.al (22) examined blood pressure in non-Caucasian subjects with DM and high blood pressure in a tropical country, and according to the results, subjects with systolic blood pressure more than 130 mmHg had eight times more chance of developing 25-hydroxyvitamin D concentration above 25 ng/ ml and five times more chance of developing 25-hydroxyvitamin D concentration above 30 ng/ ml.

Association between serum concentrations of 25-hydroxyvitamin D and blood pressure has also been studied in adolescents. According to the results, low serum concentrations of 25-hydroxyvitamin D (less than 20 ng/ml) were associated with elevated levels of diastolic blood pressure (1.09 mm Hg elevation, 95% CI: 0.04 to 2.14) in adolescents compared with normal concentrations. Systolic blood pressure tend to increase in the vitamin D deficient subjects (1.30 mm Hg elevation, 95% CI: -0.13 to 2.72). The association between systolic blood pressure and serum vitamin D concentrations was a U-shaped relationship and its relationship with diastolic blood pressure was reverse (50). At the cellular level, it has been shown that vitamin D improves endothelial function (51-53), the secretion of pro-inflammatory cytokines (54), reduces the activity of renin-angiotensin-aldosteron system and parathyroid hormone levels (55-57), and therefore, induces antihypertensive effects. Our finding suggested that there was a substantial difference regarding the components and subgroups of quality of life between the two groups (p-value <0.05), in which all the components and sub-scales were considerably lower in diabetic participants in comparison with healthy controls. Based on the findings, there was a direct association between serum concentration of 25-hydroxy vitamin D and social function and general health components of quality of life in the patients with DM Ⅱ in contrast to healthy controls. There was also positive association between serum concentration of 25-hydroxyvitamin D with physical function component and physical health subscale of quality of life in all participants. 

The association between serum concentrations of 25-hydroxyvitamin D and quality of life in diabetic patients seen in the our study is along with the conclusions of several previous studies in other disorders. For example, in a cross-sectional study, there was a direct correlation between vitamin D deficiency and quality of life in elderly women with osteoporosis and multiple disabilities (15). In the studies of diabetic patients, there was a significant association between vitamin D deficiency (25 OH D <15 ng/ml) and the quality of life (58). However, in a cross-sectional study in Dutch diabetic subjects with vitamin D deficiency with poor glycemic control, there was no correlation between the serum 25-hydroxyvitamin D concentrations and the health associated quality of life (20). It seems that the different tools used to evaluate the quality of life and the presence or absence of vitamin D deficiency in subjects lead to these inconsistencies.

Results showed that with each increasing unit in the BMI, the chances of having high systolic blood pressure will increase by 11%. Several studies have been carried out on the effects of vitamin D deficiency on obesity (59-61). It has been reported that the serum concentration of 25-hydroxyvitamin D is lower in overweight/obese subjects with a reverse relationship with the risk of obesity (62, 63). There is a link between vitamin D insufficiency and increased infiltration of fat in skeletal muscle, which can contribute to insulin resistance (64). In addition, there is some evidence of the potential role of vitamin D as an adjustable risk factor in mortality, especially cardiovascular mortality and the severity and intensity of various disabilities in patients with type 2 diabetes (65-67).

From the study limitations, we can refer to the relatively small sample size in the case (n=88) and control (n=77) groups, the impossibility of determining the causal relationship between serum concentration of 25-hydroxyvitamin D and high systolic and diastolic blood pressure due to the observational nature of the study, the lack of measurement of inflammatory markers such as CRP or ESR, and their associations with the serum 25-hydroxyvitamin D concentrations and failure to evaluate the effect of abnormal blood pressure on future blood pressure due to the need for long-term follow-up. On the other hand, despite examining several confounding variables, other undetected confounders may possibly modify the results of the present study. Despite these limitations, the present study is one of the few studies to investigate the relationship between the serum concentration of 25-hydroxyvitamin D and risk of high blood pressure in obese/overweight diabetic patients.

In conclusion** s**erum concentration of 25-hydroxyvitamin D is significantly lower in patients with DM Ⅱ than healthy subjects. There is a significant reverse relationship between serum concentration of 25-hydroxyvitamin D and diastolic blood pressure and a significant positive relationship with the components of physical and social function and general health, and the subscale of physical health of quality of life in patients with DM Ⅱ. Diabetic patients with vitamin D deficiency were 2.2 times more probable to have high diastolic blood pressure compared with those with normal vitamin D status.
